# Clinical and Radiological Results of Fixation of Acromioclavicular Joint Dislocation by Hook Plates Retained for More Than Five Months

**DOI:** 10.5812/traumamon.13728

**Published:** 2014-03-01

**Authors:** Dawood Jafary, Hassan Keihan Shokouh, Farid Najd Mazhar, Human Shariat Zadeh, Tahmineh Mochtary

**Affiliations:** 1 Department of Hand Surgery, Shafa Orthopedic Center, Iran University of Medical Sciences, Tehran, IR Iran

**Keywords:** Acromioclavicular Joint, Coracoclavicular, Ligaments

## Abstract

**Background:**

Hook plates are used to treat acromioclavicular joint dislocations. Our study took into consideration the patients’ outcome following treatment with clavicular hook plates retained for more than five months.

**Objectives:**

Our aim was to assess the response to treatment of acromioclavicular joint dislocation by clavicular hook plate when retained for more than five months.

**Patients and Methods:**

We treated 24 patients who had acromioclavicular joint dislocation with a clavicular hook plate between 2008 and 2012 at our hospital. We did not repair the coracoclavicular ligament. In all patients, the plate remained more than five months because they did not come back at the recommended time for removal of their plates. The follow-up period ranged from five to thirty three months with a mean of nineteen months.

**Results:**

The main complication was osteolysis that was seen in two patients. The mean constant score was 94.5 ± 8.77 out of 100 with a range between 70 and 100.

**Conclusions:**

Our study showed that the use of clavicular hook plates was a good treatment option for acromioclavicular joint dislocation. However, scores were lower in case of prolonged presence of plates.

## 1. Background

There are different options for treatment of acute acromioclavicular joint dislocations (1-4). These include bandages, fixation of the acromioclavicular joint with pins, tension band wiring the modified Weaver-Dunn procedure, fixation with washer and screw, and clavicular plate. All of these options have different advantages and disadvantages. So there is no clear superior modal (5, 6). However, the use of hook plates is an effective treatment option for acromioclavicular joint dislocation to improve shoulder function and allow early mobilization of the shoulder (7-10). Although there have been favorable results in many studies (9, 10), several documented complications such as infection and acromial osteolysis have been reported (7, 9, 10). Hook plates are pre-bent plates in different sizes and varying depths to fit different anatomy. A hook is placed posteriorly, with right or left sided types, and is used for distal clavicular fractures (Neer Type II) as well as acromioclavicular joint dislocations (Rockwood type III to V). It is advocated by the producers to remove the plate after three months of operation to prevent impingement and acromial osteolysis. It should also be used carefully in elderly patients. 

## 2. Objectives 

The objective of this study was to evaluate the outcome of surgical treatment of acute acromioclavicular dislocations (types III and V) using fixation with a hook plate, without coracoclavicular and acromioclavicular ligament reconstruction in patients who did not refer for removal of plates for at least five months.

## 3. Patients and Methods

**Figure 1.**
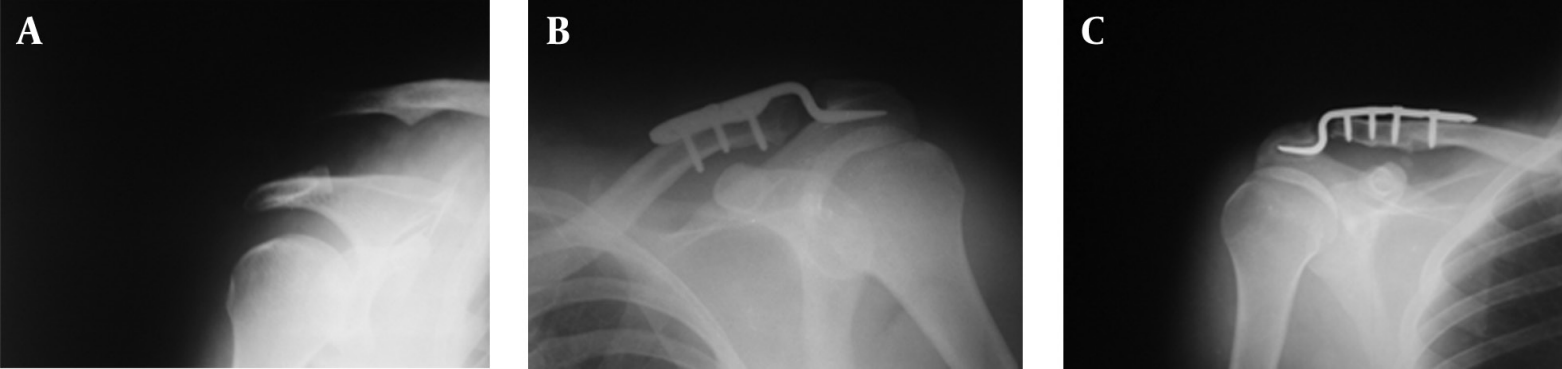
Radiographies A) Acromioclavicular joint dislocation, B) Post operative X-ray, C) Acromial osteolysis

**Figure 2.**
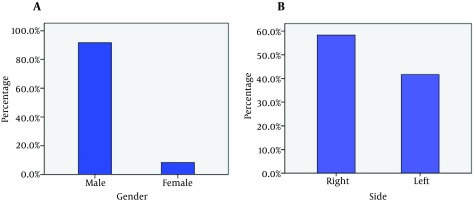
Gender Distribution of Our Patients

A total of 46 patients with acromioclavicular joint dislocation were treated with hook plate in our hospital from 2008 to 2012. Our study was a retrospective investigation. We excluded 17 patients whose plates were removed before five months from the beginning of the study. In five cases we could not contact the patients. The remaining 24 patients were included in our study. Acromioclavicular joint dislocations were treated with hook plates without repairing the coracoclavicular ligaments in these patients. In all patients, the plate remained at least for five months. Regarding the typing, four patients had Rockwood type III (16.7%) and twenty patients (83.3%) had Rockwood type V dislocations (Figure 1 A). There were two (9%) female patients and twenty two (91%) male patients (Figure 2 A). Fourteen injuries (59%) were on the right side and ten injuries (41%) were on the left (Figure 2 B). The mechanisms of injury included eight car-to-pedestrian accidents (33%), six motorcycle accidents (25%), four falls from heights (17%), and six car-to-car accidents (25%). The patients were operated by different surgeons. Operations were done in the beach chair position. A small roll was placed under the ipsilateral shoulder. An incision 5 to 7 cm in length was made along Langer’s skin lines, 2 cm medial to the acromioclavicular joint. Full thickness subcutaneous flaps were made for exposure of deltoid and trapezius aponeuroses, acromioclavicular joint, and the lateral 3 cm of the clavicle. Thereafter fascia, periosteum, and capsules were incised at the junction of the anterior one third and posterior two thirds of the clavicle to expose the dislocated joint and lateral third of the clavicle. All or part of the meniscus was removed in case of injury, and the dislocated acromioclavicular joint was reduced and fixed using an appropriate hook plate. Hook plates were stainless steel. After operation, a shoulder sling was used for comfort and range of motion therapy was started after two weeks. The mean follow-up period was nineteen months with a range from 5 to 33 months (Table 1). 

**Table 1.** Patients’ Information Based on Constant Score ^a^

No.	Age at Insertion, y	Gender	Duration of Plate Preservation, mo	Type of Trauma	X-ray Finding	Side	Type of Separation	Pain Severity(Max 5)	Activities + Positioning Score (Max 20)	Range of Motion Score (Max 40)	Power Score (Max 25)	Constant Score (Max 100)
**1**	53	Male	5	CM ^b^	N	L	3	15	20	40	25	100
**2**	30	Male	8	CP	N	R	5	15	20	40	25	100
**3**	40	Female	6	CP	N	R	3	14	18	40	22	94
**4**	48	Male	16	CP	N	R	5	13	17	30	23	82
**5**	42	Male	5	CP	N	R	5	15	20	40	25	100
**6**	18	Male	9	CM	N	L	5	15	20	40	25	100
**7**	33	Male	8	CM	N	L	5	14	18	40	24	96
**8**	51	Male	7	Falling	N	R	5	15	20	40	25	100
**9**	67	Male	33	Falling	Ost	R	5	12	16	22	20	70
**10**	24	Male	10	CM	N	L	5	14	18	40	24	96
**11**	23	Male	8	CC	N	R	3	15	20	40	25	100
**12**	33	Male	12	CC	N	L	5	14	17	38	23	92
**13**	49	Male	5	CC	N	R	5	15	20	40	25	100
**14**	24	Male	6	CC	N	R	5	15	20	40	25	100
**15**	23	Male	9	Falling	N	L	5	15	20	40	25	100
**16**	31	Male	11	CM	N	R	5	15	20	40	25	100
**17**	49	Male	13	CC	N	R	3	12	13	34	19	88
**18**	65	Female	18	CP	Ost	R	5	13	15	26	20	74
**19**	26	Male	7	Falling	N	L	5	15	20	40	25	100
**20**	51	Male	6	CP	N	L	5	15	20	40	25	100
**21**	40	Male	8	CC	N	R	5	15	20	38	25	100
**22**	57	Male	14	CM	N	L	5	13	12	36	23	84
**23**	47	Male	6	CP	N	L	5	14	19	38	22	92
**24**	44	Male	6	CP	N	R	5	15	20	40	25	100

^a^ Statistical analysis was performed using SPSS for Windows, Version 16.0 (Chicago, SPSS Inc.).

^b^ Abbreviations: CC, car-to-car accident; CM, car-to-motorcycle accident; CP, car-to-pedestrian accident; L, left; Max, maximum; N, normal; Ost, osteolysis; R, right.

**Table 2.** The Mean Constant Score Consisting of Pain Severity, Range of Motion, Activities, Positioning, and Power, (Maximum Score of 100) ^a^

Pain Score	Activities + Positioning Score	Range of Motion Score	Power Score	Constant Score
**14.29 ± 0.9**	18.45 ± 2.35	37.58 ± 4.86	23.75 ± 1.87	94.5 ± 8.77

^a^ Data are pesented as mean ± SD.

## 4. Results

Our patients did not experience infection or plate failure during this study. We used a constant score for pain severity (15), range of motion (40), forward flexion (10), abduction (10), internal rotation (10), external rotation (10) of shoulder, activity level (20), patients’ condition regarding sleep (2), exercise (4), work (4), positioning in space (10), and power (25) with a total score of 100. The mean score pain severity was 14.29 ± 0.9 out of 15 (range 12 - 15). The mean score of activities and positioning in space was 18.45 ± 2.35 out of 20 (range 12 - 20). The mean score of range of motion was 37.58 ± 4.86 out of 40 (range 22 - 40). The mean power score was 23.75 ± 1.87 out of 25 (range 19 - 25), and the mean of constant score was 94.5 ± 8.77 out of 100 (Table 2). The minimum score was 70 and the maximum score was 100. We had ten patients with a score less than 100. Five of them had scores below 90. The X-ray findings were normal in all patients (Figure 1 B) except two of them who developed acromial osteolysis (Table 1). One patient was an old man whose plate could not be removed due to cardiopulmonary problems. He showed osteolysis of acromion at X-ray examination (Figure 1 C). His constant score was 70, the lowest score. The other patient who had osteolysis was a 65-year-old female and her plate had remained for 18 months. She had a total score of 74. The other three patients with scores under 90 did not have osteolysis in their X-ray, but had pain when lying on the operated shoulder and at the time of heavy activities. Moreover, their shoulder range of motion was limited. In fourteen patients, the shoulder performance was good and the constant score was excellent. 

## 5. Discussion

 Many operations and different types of devices have been used to treat acromioclavicular dislocations with different outcomes (1, 3-6, 9). One of the techniques that has been proven to be an effective treatment of acromioclavicular joint dislocation is using a clavicle hook plate (1, 6, 8). We tried to use this method for restoration of biomechanics of the shoulder girdle and the results appeared to be satisfactory even when the plates were in situ more than five months. Patients whose plates remained more than 12 months had more complaints. Although some authors did not find it necessary to remove the implant (3), these patients complained from pain during sleep and some of them were unable to lie on the operated side. This dissatisfaction was present even in patients with a good range of motion. There is a general recommendation that the implant should be removed at around three months after operation. In most of our patients there was a lack of interest to remove the plate because they felt it was unnecessary, but later, they all came back to remove it. Only in two patients was osteolysis seen on follow-up X-rays. In both cases, the plate remained more than 18 months. There was no clavicular fracture at the medial end of the retained implant despite the report of these fractures in the literature (8). Impairment of shoulder movement was reported following other types of operations due to metal devices used to fix the acromioclavicular joint. This complication was present in all of our patients when the plate remained more than 12 months (10). Because of the small number of our patients, we were not able to do a complete statistical analysis on the data we collected. Therefore, no particular pattern was found to predict which patients will have lower constant scores ultimately. The use of hook plate for treatment of acromioclavicular joint dislocations has been proved to be a good option. Although our patients with plates less than 12 months had good scores and X-ray findings, we recommend using the plate in patients for whom the medical condition will permit the removal of the plate. It is important to inform the patients about the necessity of timely removal of the plates as recommended to limit the morbidity associated with the plate being left in situ. 
